# Modes of hole formation in long-lasting insecticidal nets (LLINs) retrieved from South Eastern Ghana

**DOI:** 10.1186/s13071-014-0547-x

**Published:** 2014-12-05

**Authors:** Sabine K Käse, Stephen J Russell

**Affiliations:** Friedrich-Maag-Str. 18, 72458 Albstadt, Germany; Nonwovens Innovation & Research Institute Ltd, Centre for Technical Textiles, School of Design, University of Leeds, Woodhouse Lane, Leeds, LS2 9JT West Yorkshire UK

**Keywords:** Insecticide-treated bednets, Mosquito nets, Damage, Defect, Holes

## Abstract

**Background:**

Long-lasting insecticidal nets (LLINs) are expected to provide biological efficacy for at least three years in the field and be sufficiently durable to maintain physical protection. Unfortunately, LLINs structurally deteriorate during use accumulating holes. Hitherto, definitive identification of the causes of hole formation has been difficult based upon qualitative surveys.

**Methods:**

In this preliminary study, optical and scanning electron microscopy of damage in used polyester (PET) and polyethylene (PE) LLINs randomly collected via a household survey from South Eastern Ghana (n =100) were utilised to identify the cause of individual holes.

**Results:**

Multiple damage mechanisms were identified. In both PET and PE LLINs, the majority of holes were initiated by filament fracture (ductile failure and cutting) and thermal damage.

**Conclusions:**

No strong correlation was found between the bursting strength of retrieved LLINs and overall hole frequency in either the PET or PE nets suggesting that bursting strength is an unreliable predictor of resistance to hole formation if used as a sole parameter.

## Background

According to the World Health Organization (WHO) an estimated 225 million cases of malaria occurred in 106 malaria-endemic countries in 2010 representing a reduction from 244 million cases estimated in 2005 [1]. Insecticide treated nets are one of the most efficient and broadly applied tools for controlling and preventing insect vector-borne diseases such as malaria [1-3]. They are recommended by the WHO as a means of providing personal protection against human-vector contact and reducing the lifespan of female mosquitoes to minimize malaria infections amongst people at risk [1].

Between 2008 and 2010 a total of 254 million LLINs were delivered to sub-Saharan Africa, representing about 66% of the 765 million people at risk [1]. Worldwide over US $ 500 million was spent on LLINs to meet these targets representing the largest single item used for malaria vector control [4]. To achieve high malaria prevention in target areas the WHO recommends a high level distribution of LLINs to persons at risk to ensure universal coverage [1,2]. These nets are designed to maintain their biological efficacy for twenty standard laboratory washes and a minimum of three years of recommended use in the field [1,2,5].

LLINs commonly comprise warp-knitted fabrics made from continuous filament yarns composed either of polyester (PET), polyethylene (PE) or polypropylene (PP). The net fabric must maintain its structural integrity for the duration of its insecticidal efficacy to provide a physical barrier against mosquito populations. The number and size of holes in nets gradually increases with time of use [6-8], and this accumulated physical damage is a common reason for households to discard nets [7,9,10]. Resistance to hole formation is therefore important to increase long-term physical barrier performance as well as user acceptance over an extended period. Several studies, including those by Githinji et al. [11], Banek et al. [12], Tami et al. [13], Asidi [14] and the WHO Pesticide Evaluation Scheme (WHOPES) [15], have been conducted to analyze the insecticidal durability, and number and size of holes in used mosquito nets collected from the field. Skovmand and Bosselmann [16] have also reported on how the structure and composition of LLINs can affect their durability. The WHO has provided guidance for vector control programmes to measure the durability of LLINs in the field including analysis of the fabric integrity [2,4].

The durability of nets may be dependent upon various factors, such as climate, conditions and frequency of use including location (indoors or outdoors), living standards, washing frequency, presence of rodents and other animals [2]. Currently, to characterize the mechanical robustness of new LLINs fabric bursting strength is determined according to standard methods [17,18]. Bursting strength can be defined as the multidirectional resistance to rupture of a circular fabric specimen [19] and new LLINs are expected to achieve a minimum value of 250 kPa [20].

The integrity of used LLINs is measured by assessing the number, location and size of holes. It is also recommended to categorize the type of hole and to indicate the likely cause of hole formation, e.g. by burning, tearing, seam failure or being nibbled or chewed by animals [2]. However, this categorization is typically based on visual inspection of nets and surveys rather than direct microscopic analysis of individual defects and hole morphologies.

Given the many different sources of wear and tear that LLINs are likely to be subjected to in the field, it is instructive to develop a detailed understanding of real modes of LLIN damage that occur, preferably by direct analysis of accumulated defects. Accordingly, the aims of this preliminary study were to microscopically analyse the morphologies of all individual defects and holes in a small sample of used LLINs retrieved from the field to positively identify the nature of the physical damage and to determine if this was correlated with their initial bursting strength.

## Methods

### Study and sample collection

Used LLINs were randomly collected via a household survey from several locations of the same region of Ghana, including the towns of Woe, Keta and Agbozume (collected and supplied by Vestergaard Frandsen). The products were manufactured from 2004 to 2008 and were in use until June 2010. The distribution dates of the LLINs were not known but the majority would have been subjected to numerous potential sources of damage enabling multiple defects to be accumulated and the focus of this study was the characterization of types of damage rather than quantitative comparison of the numbers or sizes of holes between brands.

The collected LLINs comprised two of the most commonly utilized products made from multifilament PET yarn (n = 52; PermaNet® 2.0) and monofilament PE yarn (n = 48; Olyset® Net). In the PET LLIN sample, fifty comprised of 75 denier yarns and two of 100 denier yarns. These PET and PE LLINs differed not only in their polymer composition and insecticide technology but also in yarn linear density, yarn construction (multifilament in the case of PermaNet® 2.0 and monofilament in the case of Olyset®) as well as knitting pattern.

### Sample analysis

**Figure 1:**
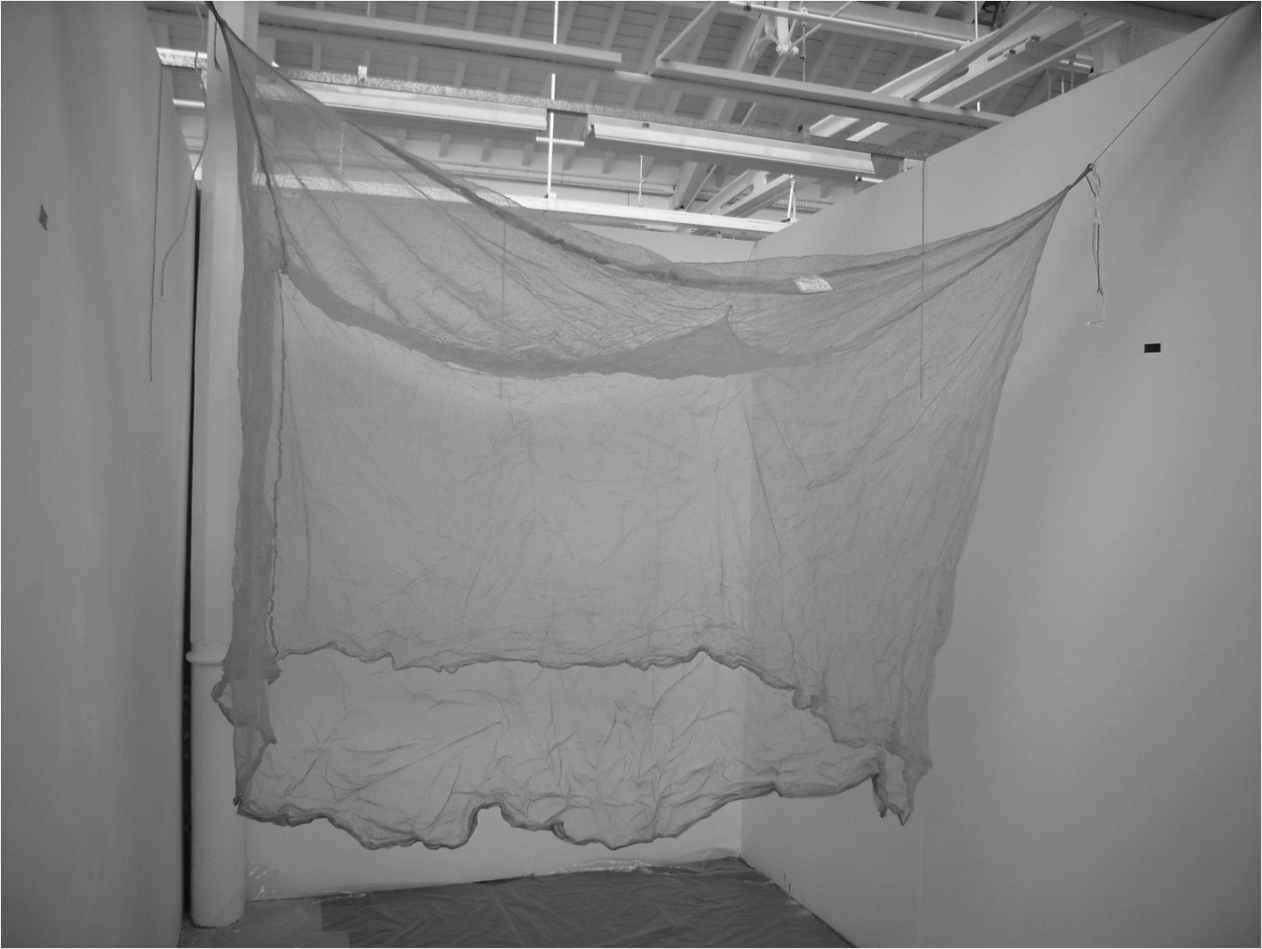
**Suspended LLIN for identification of damage.**

To aid the location of individual defects, LLIN specimens were suspended at each corner (Figure 1) and examined within the area contained within the continuous 1 m strip from the base of the net. Defects have previously been found to be most prevalent in this location [21,22].

Optical microscopy was utilised to inspect modifications in fabric structure such as knitted loop distortions and protruding filaments upon the fabric surface with sizes as small as 1 mm. Scanning Electron Microscopy (SEM) was employed to inspect broken filament ends and any modifications to the sectional and longitudinal filament morphologies (μm-scale). For the purpose of categorization, holes were defined as defects in which one or more adjacent yarn severances had occurred. Yarn in this context means the entire multifilament (PET) or the monofilament (PE) yarn.

Based on the microscopic evidence, the mode of structural damage could be elucidated for each individual defect guided by characteristic forms of filament and fabric structural damage that has been described in the literature [23-27]. This involves analysing the morphology of each defect around and within its perimeter in the fabric and the fracture faces of severed filaments. Small holes in LLINs caused by the severance of just a few yarns can sometimes propagate in to larger ones by unravelling of the knitted structure, and these have a very characteristic appearance, which is easy to identify. The focus in this work was on damage associated with severed yarns in the region of a hole, rather than on unravelling defects, which can occur afterwards.

### Data analysis

Statistical analysis of the data (OriginPro 8.1 SR1 Version 8.1.13.88) was performed using linear regression. The correlation coefficient was calculated to determine the strength of the association between defect types, number of holes and bursting strength of the used LLINs.

### Fabric bursting strength

The bursting strength of each used net was determined based on ISO 13938-1 methodology using at least three replications per sample [17]. Test specimens were prepared using regions that did not contain holes as defined herein.

## Results

### Identification of structural damage

**Figure 2:**
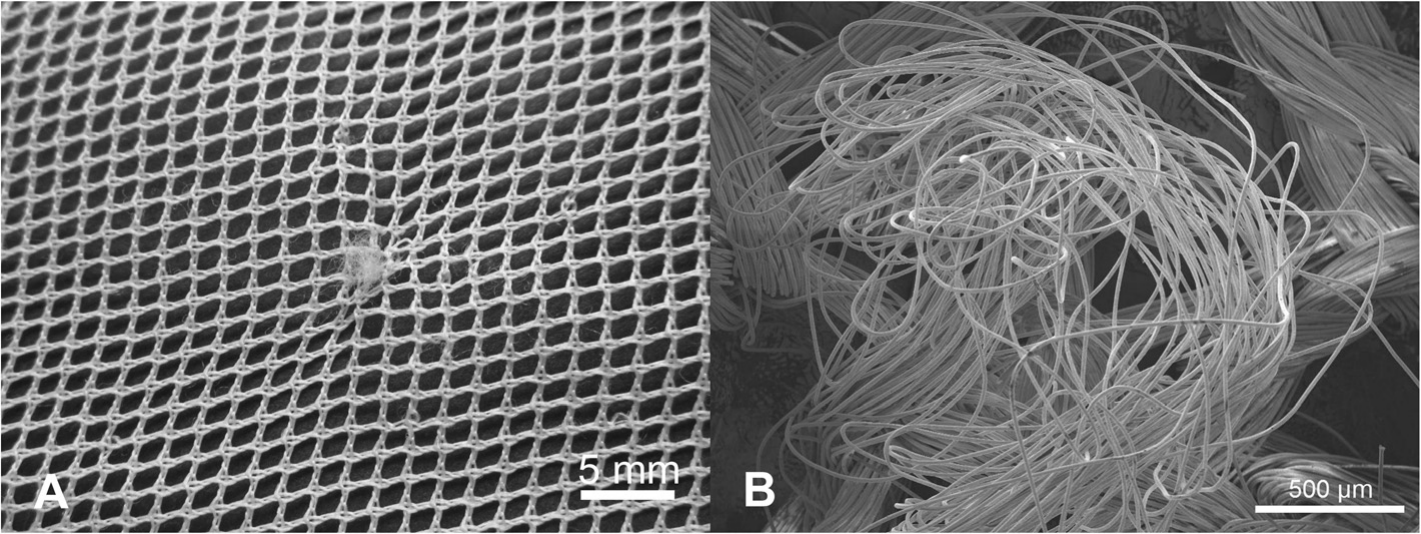
**Snags in PET LLINs. (A)** Appearance of a typical snag defect showing localized distortion of fabric and protruding filaments; **(B)** Displaced filaments within a snag.

**Figure 3:**
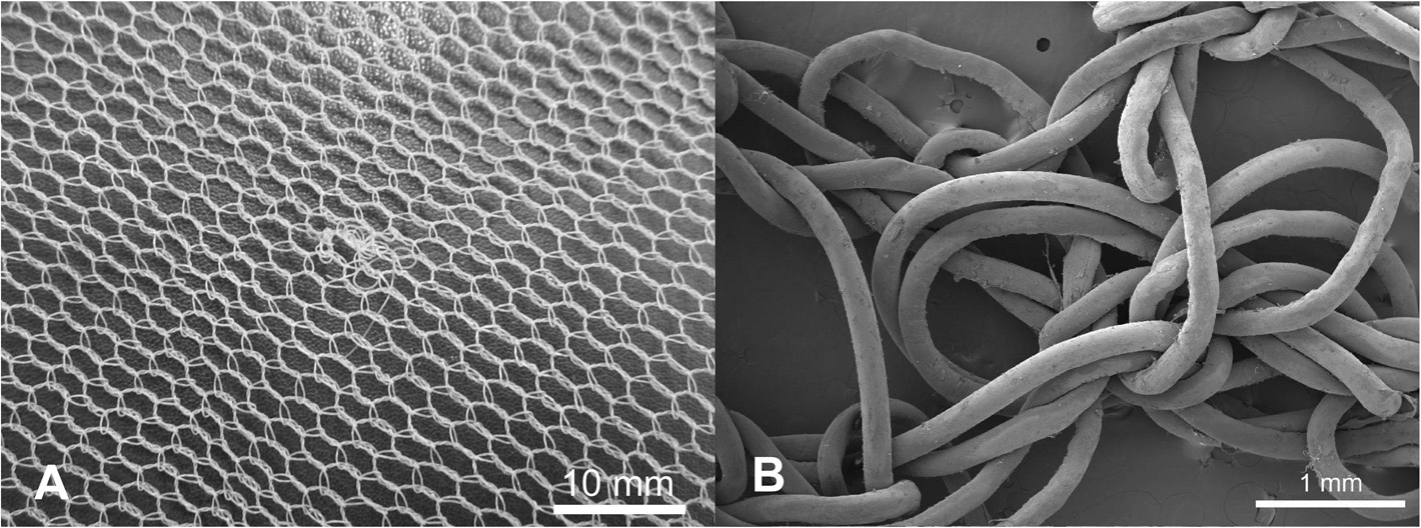
**Snags in PE LLINs. (A)** Example of a snag in a PE LLIN, and **(B)** displaced PE monofilaments due to snagging.

**Figure 4:**
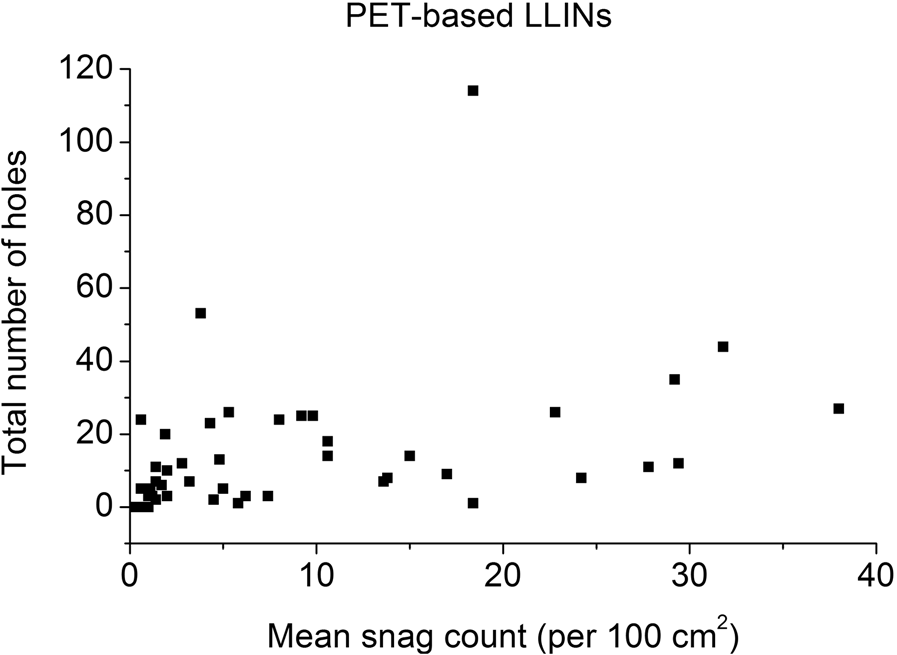
**Mean snag count vs. total number of holes in used PET LLINs.**

Damage in the form of snags was observed in both the PET and PE nets (Figures 2A and 3A). Typically, these appeared as filament protrusions, most commonly in the form of loops and distortions in the net structure as well as filamentation caused by broken yarn filaments (Figures 2B and 3B). Snag damage is produced when a rough or sharp object pulls, plucks or drags a group of filaments or a yarn segment from its normal position in a knitted fabric, leading to distortion and/or protruding filaments [28]. Practically, this may result from the fabric being caught on an angular, jagged or sharp projection. Note that a snag is not necessarily associated with a hole since the filaments within the defect may remain unbroken. The PET LLINs were found to contain up to 38 snags/100 cm^2^ whereas the mean snag count in the PE sample was smaller (0.01 snags/100 cm^2^). Whilst a snag initially involves distortion of the net structure with or without filament severances it is possible that a hole will be created after snagging if the projecting yarns are subsequently broken. The potential association between the frequency of snags and hole formation in the same sample of PET nets was explored by regression analysis (Figure 4), but a strong correlation could not be confirmed (r^2^ = 0.17).

**Figure 5:**
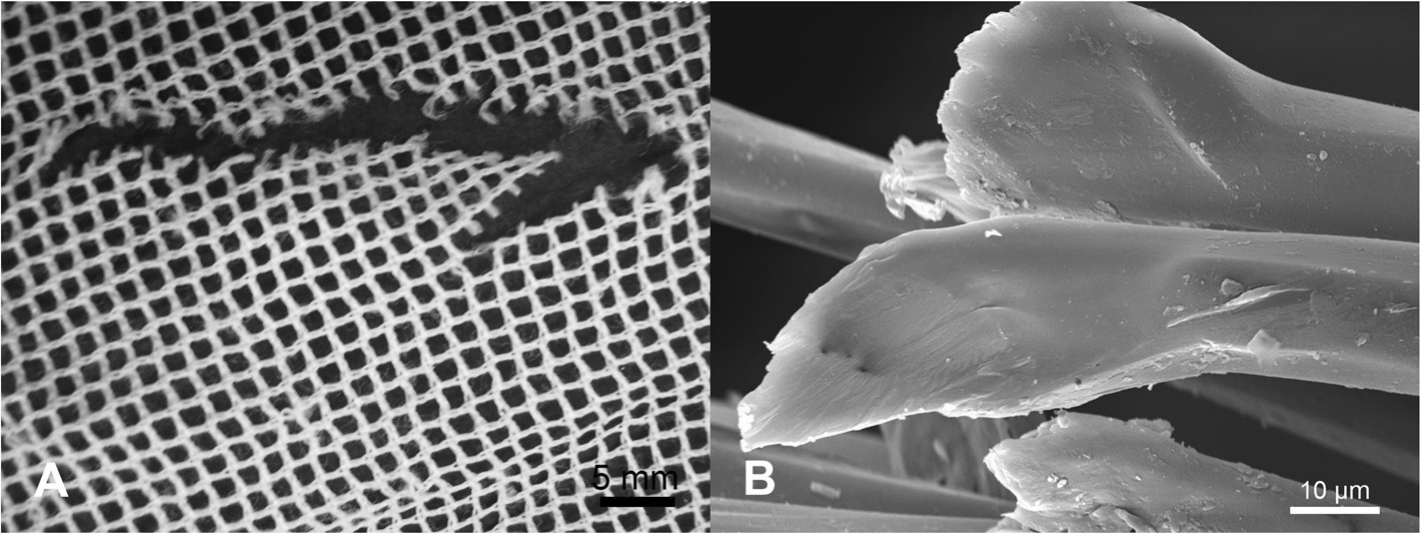
**Hole formation by filament cutting in PET LLINs. (A)** Example of a hole formed by cutting of filaments in a PET LLIN and **(B)** cut PET filament ends.

**Figure 6:**
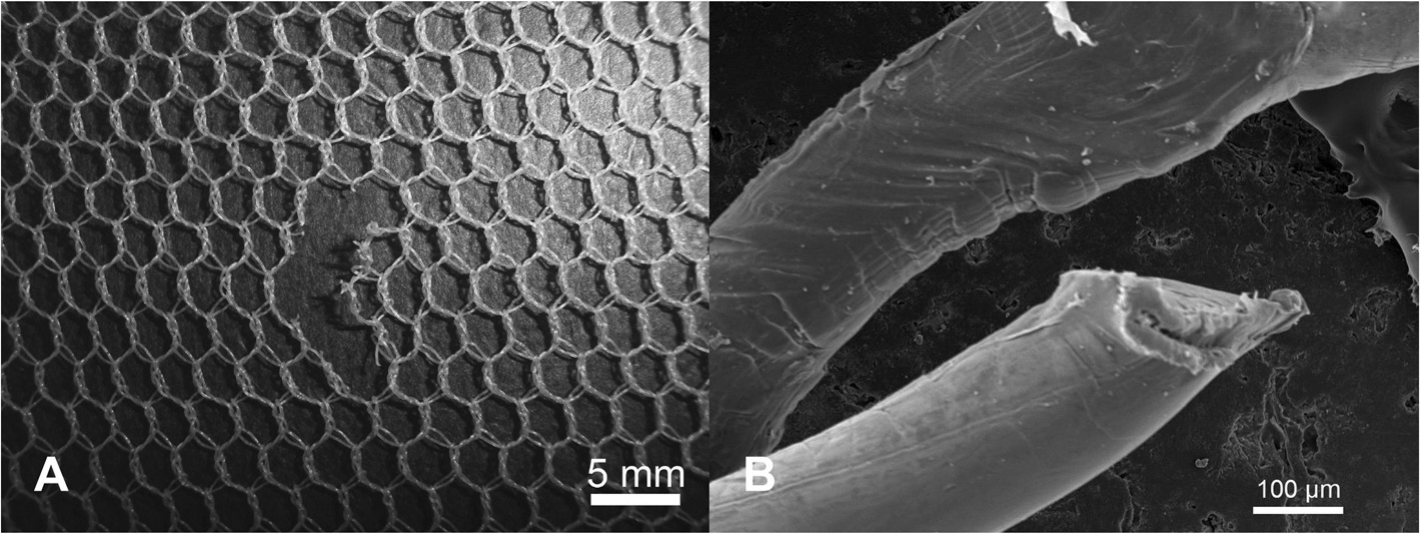
**Hole formation by filament cutting in PE LLINs. (A)** Example of a hole formed by cutting of filaments in a PE LLIN and **(B)** cut PE filament end.

**Figure 7:**
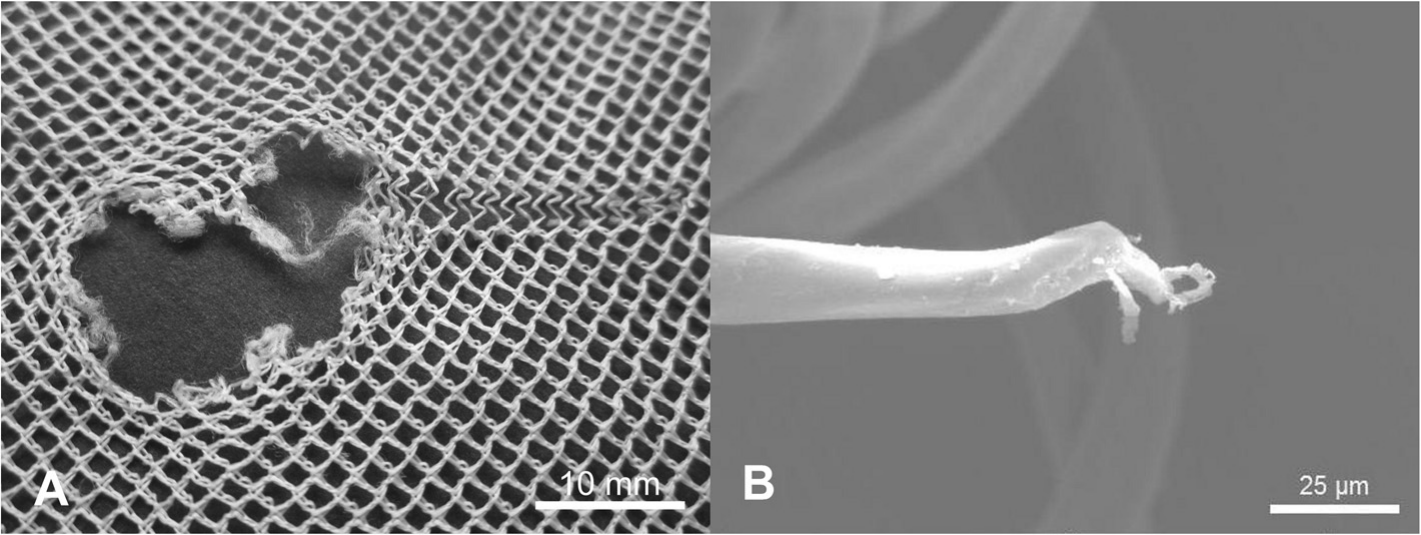
**Hole formation by filament rupture and tear propagation in PET LLINs. (A)** Example of hole formed by filament rupture and tear propagation in a PET LLIN; **(B)** Ruptured PET filament end located within the perimeter of a torn region.

**Figure 8:**
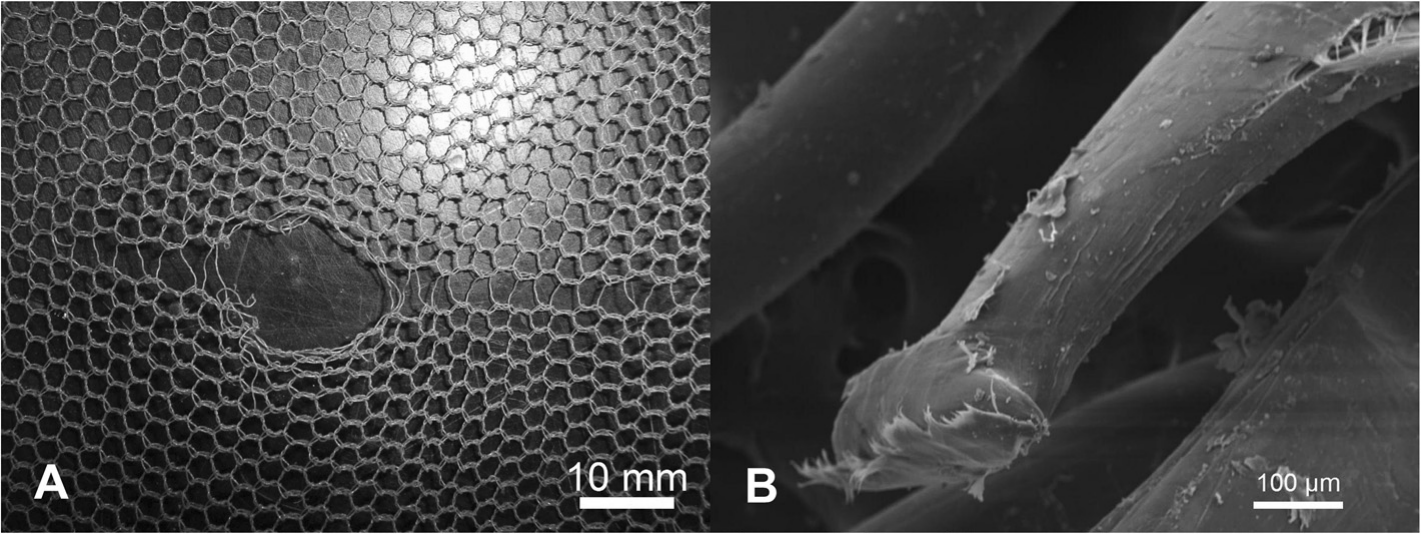
**Hole formation by filament rupture and tear propagation in PE LLINs. (A)** Example of a hole formed by filament rupture and tear propagation in a PE LLIN; **(B)** Ruptured PE filament end located within the perimeter of a torn region.

**Figure 9:**
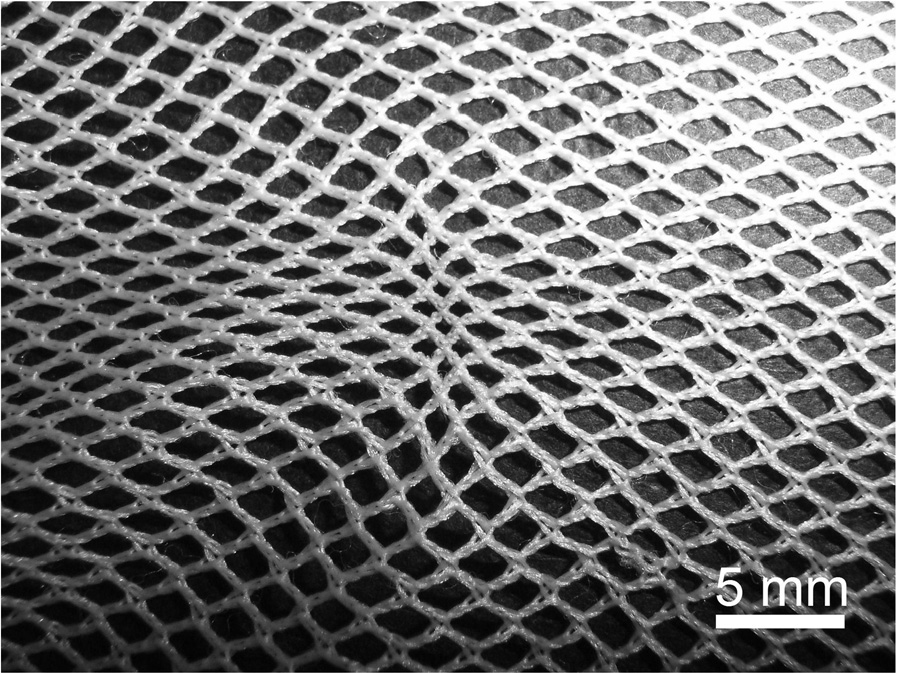
**Damage caused by thermal shrinkage in a PET LLIN.**

**Figure 10:**
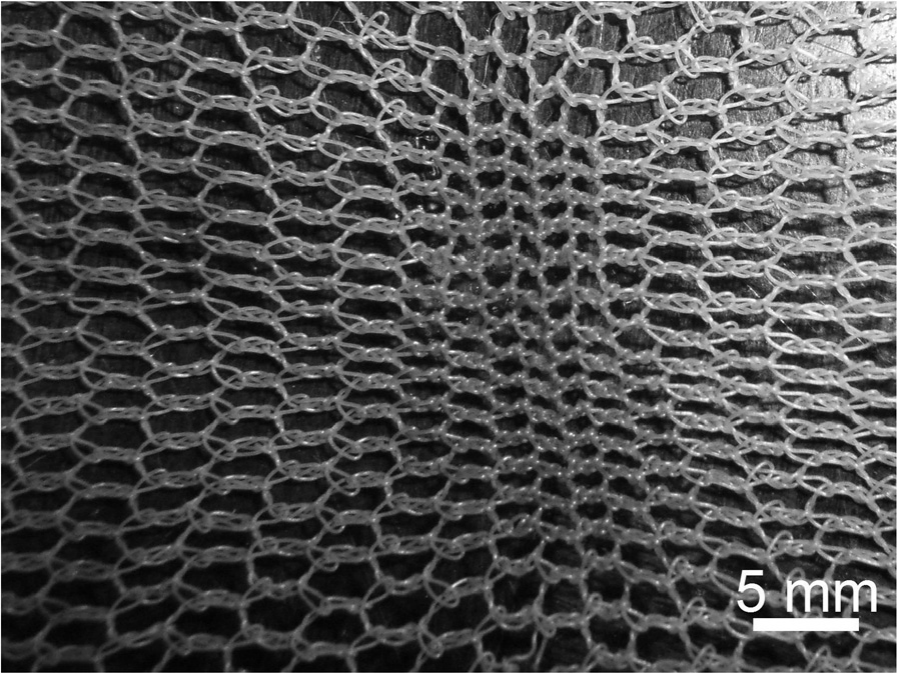
**Damage caused by thermal shrinkage in a PE LLIN.**

**Figure 11:**
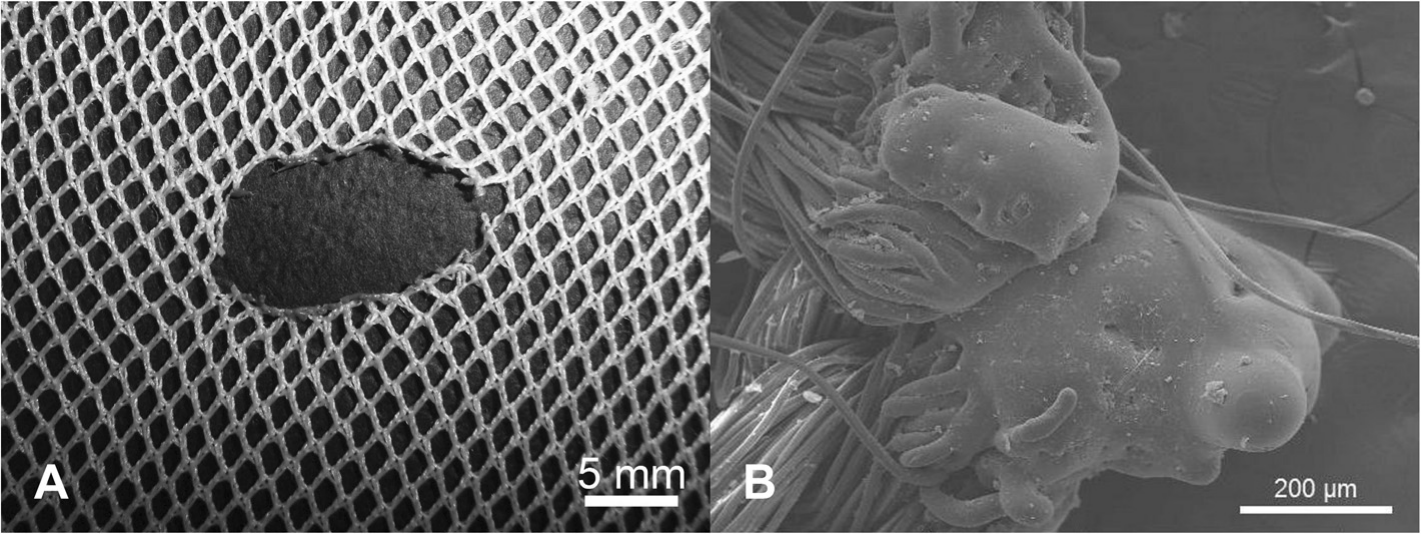
**Hole formation by thermal damage in PET LLINs. (A)** Hole resulting from thermal damage (polymer melting) in a PET LLIN and **(B)** PET filament damage caused by melting.

**Figure 12:**
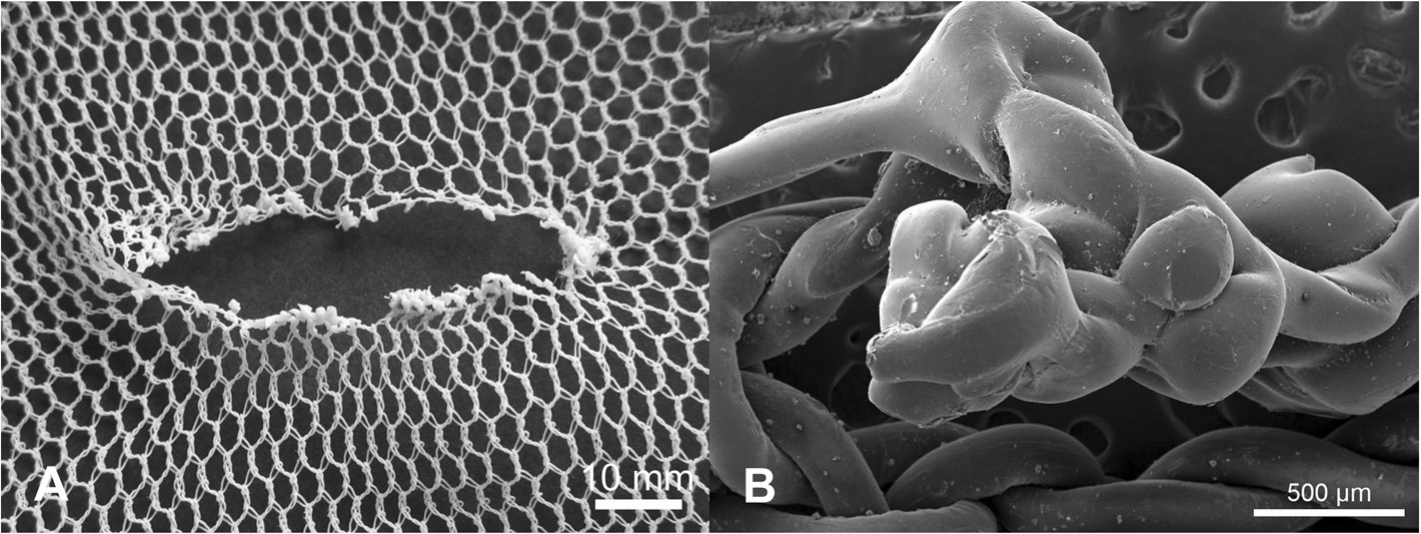
**Hole formation by thermal damage in PE LLINs. (A)** Hole formed by thermal damage and melting of PE in a LLIN, **(B)** PE filament damage resulting from melting of the polymer.

SEM of filament ends in the region of individual holes revealed a variety of different damage morphologies, see for example Figures 5A, 6A, 7A and 8A, and different modes of damage, were identified as follows:

- Puncture and propagation as a result of sharp transverse pressure – smooth disjoined filament ends produced by cutting of isolated filaments (Figures 5B and 6B) with minimal distortion of the surrounding knitted structure (Figures 5A and 6A).
- Puncture and propagation as a result of blunt transverse pressure – ruptured and distorted broken filament ends associated with the effects of ductile fracture as well as shear and lateral forces applied across the filament cross-section (Figures 7A and 8A) together with directional distortion of the knitted structure (Figures 7B and 8B). These features were consistent with tearing where filaments break and a hole is formed as a solid object penetrates the structure and the fabric is pulled apart forcefully. This could arise if the net was caught upon an external object and then either one being moved relative to the other. In these circumstances, the fabric was directionally tensioned as a result of physical contact with the external object.
- Melting and plastic flow as a result of contact or proximity to a high temperature source – thermal shrinkage of yarns leading to localised distortion of the fabric structure (Figures 9 and 10) as well as hole formation due to polymer melting were evident as a result of heat exposure (Figures 11A and 12A). Exposure to a temperature above the polymer melting point gave rise to melt flow and formed a hole with characteristic filament end morphology within its perimeter (Figures 11B and 12B).

### Frequency by damage category

**Figure 13:**
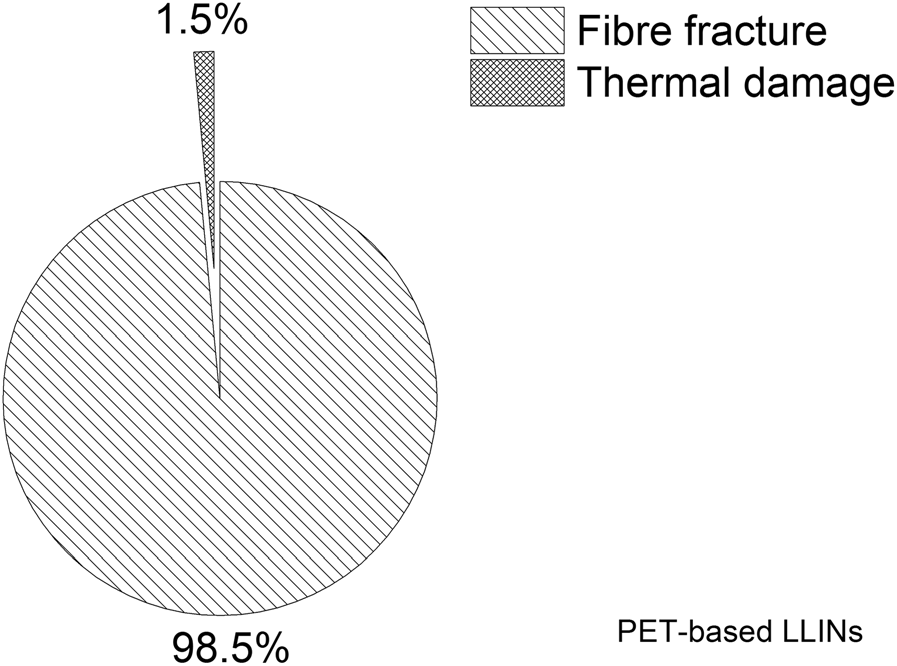
**Distribution of hole forming mechanisms in PET LLINs.**

**Figure 14:**
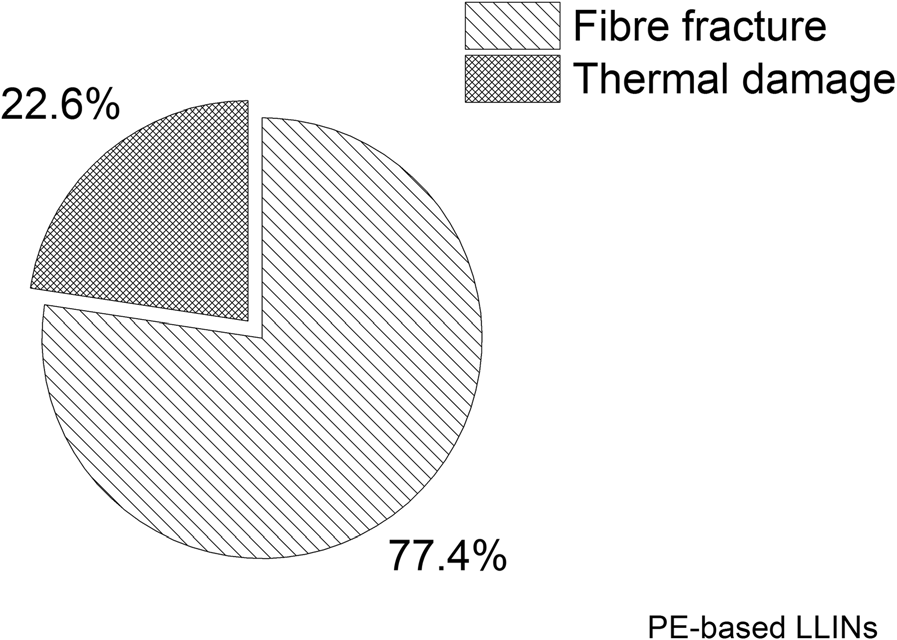
**Distribution of hole forming mechanisms in PE LLINs.**

The number of holes associated with mechanical damage (cutting and ductile fracture) and thermal damage (melting) [23] was determined for the PET (Figure 13) and PE LLINs (Figure 14). The majority of holes in both the PET (98.5%) and PE (77.4%) samples were found to be associated with ductile fracture due to localized mechanical stress. Damage associated with thermal damage was 1.5% and 22.6% for the PET and PE samples respectively.

**Figure 15:**
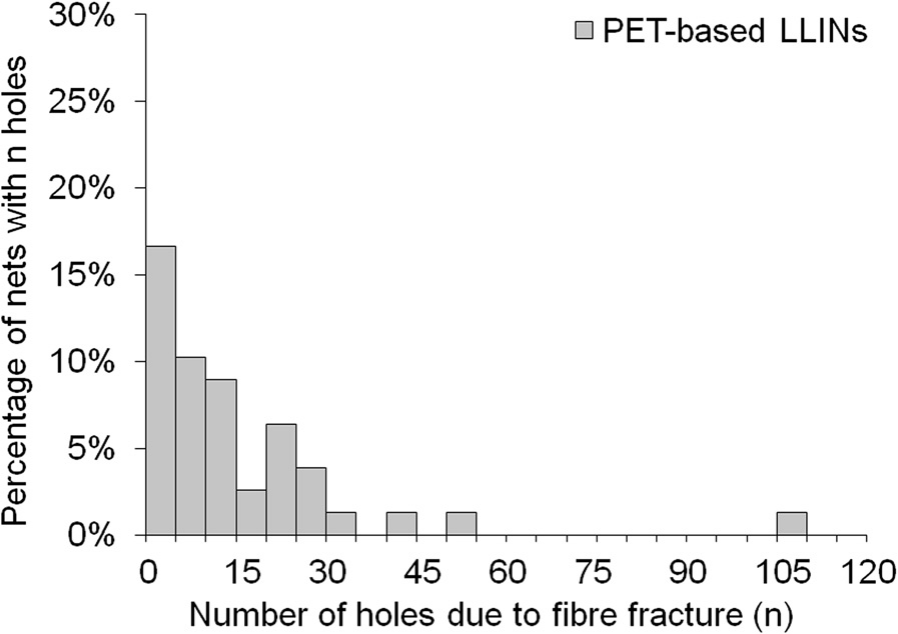
**Distribution of the number of holes associated with fibre fracture in PET LLINs.**

**Figure 16:**
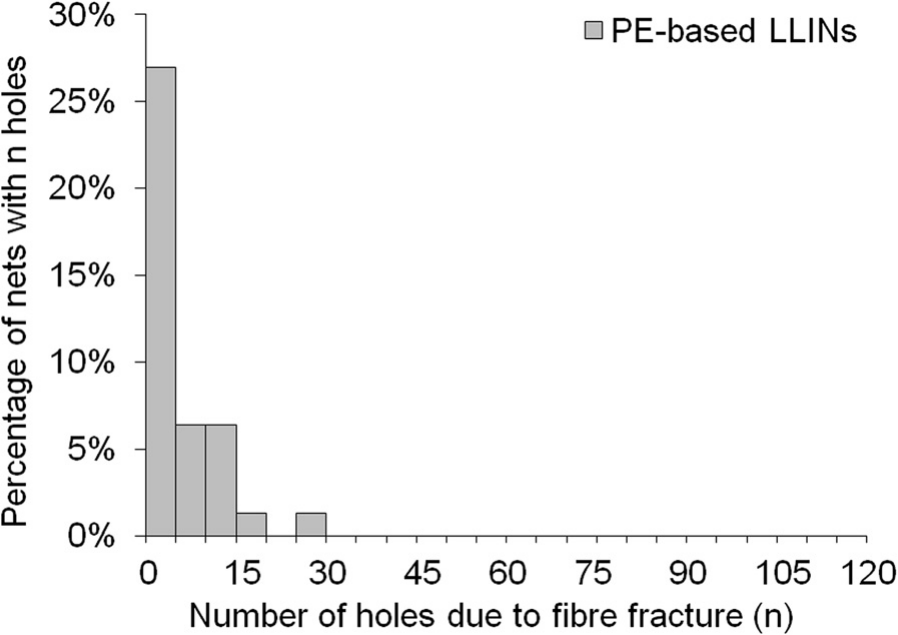
**Distribution of the number of holes associated with fibre fracture in PE LLINs.**

**Figure 17:**
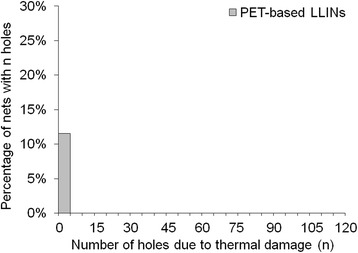
**Distribution of the number of holes associated with thermal damage in PET LLINs.**

**Figure 18:**
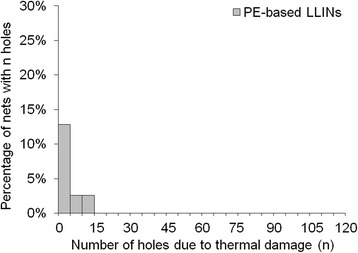
**Distribution of the number of holes associated with thermal damage in PE LLINs.**

In terms of damage frequency per net, a large proportion of LLINs had less than five holes per net due to fibre fracture (Figures 15 and 16) and thermal damage (Figures 17 and 18). However, a small number of the PET LLINs exhibited thirty or more holes associated with fibre fracture per net (Figure 15) but for the PE LLINs the number never exceeded thirty (Figure 16). The total frequency of holes per net attributable to thermal damage never exceeded two in the PET LLINs (Figure 17) but was thirteen in one PE LLIN (Figure 18).

### Bursting strength of used LLINs

**Figure 19:**
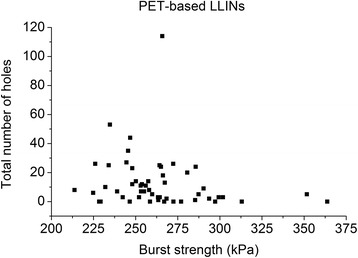
**Fabric bursting strength vs. total number of holes in used PET LLINs.**

**Figure 20:**
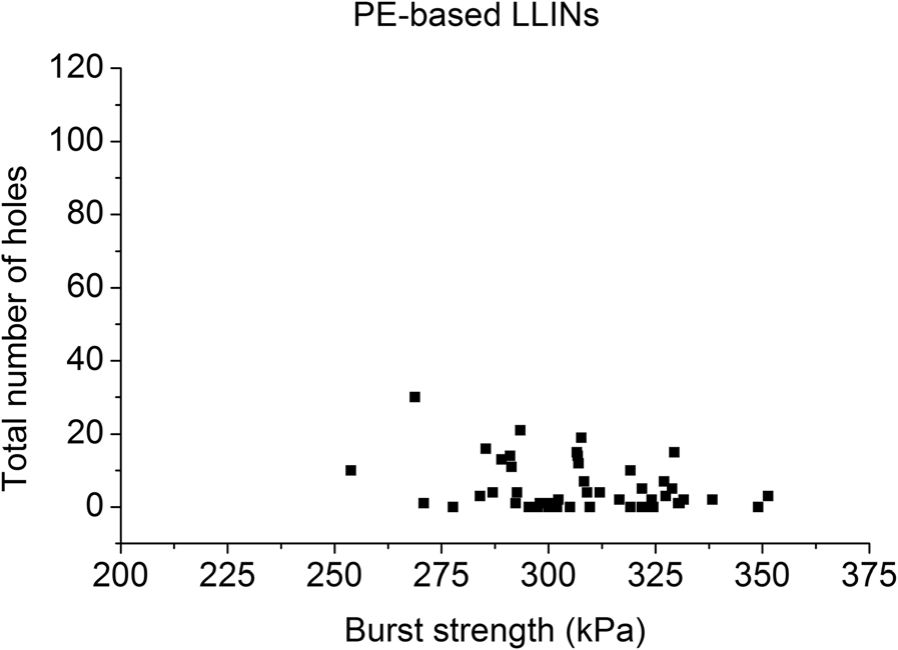
**Fabric bursting strength vs. total number of holes in used PE LLINs.**

The bursting strength of the used PET LLINs ranged from 210–320 kPa for LLINs made from 75 denier yarns and 350–370 kPa for LLINs from 100 denier yarns (Figure 19). For PE LLINs knitted from 150 denier yarn, the bursting strength ranged from 250–360 kPa (Figure 20). The bursting strength values of some of the used PET specimens were below the recommended 250 kPa threshold for new LLINs [20], suggesting that fabric properties may be affected by weathering associated with long-term, continuous use in the field prior to collection. No strong correlation between the bursting strength of used LLINs and the number of holes in the same samples was evident for either the PET (r^2^ = 0.04) or PE LLINs (r^2^ = 0.10).

## Discussion

It is clear from the microscopic evidence that more than one mechanism is responsible for the initial breakage of filaments in LLINs. These initial filament breakages may immediately produce a large hole or may provide a seed for the formation of a larger hole due to a secondary mechanism such as laddering or unravelling, as a result of continued exposure to agencies of wear.

In this sample of LLINs the basic damage mechanisms, associated with filament breakage and holes included ductile fracture, cutting and melting. Ductile fracture and cutting are principally associated with the effects of mechanical stress acting upon the filaments in the net and practically, may be introduced in various ways. LLINs are exposed to localised mechanical stress when in situ as a result of the fabric structure being snagged on sharp or rough objects in the immediate environment, possibly during hand washing [29] or as suggested by some researchers, during rodent interaction [22]. Furthermore, multiple mechanical stress events can be expected during the life of a net such that damage will be progressively accumulated. The specific appearance of mechanical stress defects arises from the initial contact and relative motion of the net’s surface with the external object. Two mechanisms involved in fibre fracture have been previously classified as surface cutting and direct frictional wear [30]. Both mechanisms can lead to fabric damage at the point of contact due to localized stress and frictional forces acting on the filaments, which may eventually lead to their fracture. Filaments can be abraded whilst firmly held within the net structure resulting in fracture, slippage or vertical displacement. Surface cutting occurs when the projections on an object are sharp and small relative to the filament diameter. In filaments severed by cutting, characteristically smooth surfaced, cleaved filament ends could be observed during SEM analysis (Figures 5B and 6B) together with an undistorted knitted structure immediately adjacent to the defect (Figures 5A and 6A). Breakage of filament ends as a result of rodent interaction is also feasible as a result of a similar mechanism, but in the present study, sub-categorisation of the cutting damage present in the LLINs was not investigated.

In comparison to cutting damage, the morphology of filament ends subjected to blunt puncture and propagation exhibit irregular and scattered fibre ends with evidence of shear and lateral force having been applied to the cross-section (Figures 7B and 8B). This may be visualized as surface cutting using a blunt object [23] under tension. As the fabric is pierced, filaments are broken by a blunt object rather than cleaved in a smooth line resulting in distorted filament ends after failure. In the present study, the majority of tears was found within the net extremities and was not propagated from the edges of the net. Tears are produced by initiation of a structural discontinuity, such as a yarn breakage (Figures 7A and 8A), followed by successive filament breakages, leading to the formation of large holes [31].

To minimize hole formation as a result of fibre fracture, resistance to initial filament breakage as well as the successive breakage of filaments is particularly important after the fabric has been initially punctured or snagged on a solid object. Successive filament breakage quickly leads to the formation of a hole large enough to undermine the physical barrier that is provided by the fabric to the passage of mosquitoes. In practice, the extent to which large tears can be resisted will be influenced by factors such as polymer composition and filament tensile properties, yarn construction, knitting pattern (geometrical arrangement and intermeshing of the constituent yarns) and surface coatings.

Fabric bursting strength is used as an indicator of LLIN durability and normally forms part of quantitative performance specifications. The lack of correlation between the bursting strength and the number of holes in used PET and PE LLINs (Figures 19 and 20) suggests that while it is universally accepted as a method of characterizing the strength of a knitted fabric, the bursting strength does not necessarily reflect the resistance of the net to the formation of holes if considered as a sole parameter. This was observed even though the majority of holes in this study were found to be the result of mechanical damage.

An important mechanism associated with damage due to mechanical stress in LLINs is snagging. Snags result in the protrusion of filaments or yarns from the fabric and/or distortion of the fabric structure [28]. Snag initiation takes place during biaxial deformation of the fabric when an asperity on a rough surface of an object plucks a segment of a filament from the fabric surface (Figures 2A and 3A). The initiated snag is then propagated when a plucked loop is distended or if it is caught and breaks [30]. Snagging propensity is related to filament yarn and fabric construction, and LLINs are conventionally made of knitted fabric structures. Knitted fabrics are known to be more susceptible to snagging because of their intermeshed construction and dimensional instability under tension [30]. Snags were particularly prevalent in the PET LLINs all of which were constructed from multifilament yarns (Figure 4), whereas the PE LLINs comprised monofilaments. Whilst LLINs accumulate snag defects and these entities constitute visible structural damage in the fabric, they are not necessarily always associated with hole formation since yarns can remain intact (Figures 2B and 3B).

Melting of the polymer was also found to give rise to holes as a result of filament damage. Net fabrics, and especially free-hanging mosquito nets, are accessible to thermal damage by such means as naked flames due to their open construction and low area density [27,32,33]. Thermal damage accounted for 1.5% of the total number of observed holes in the PET LLINs (Figure 13) and 22.6% in the PE LLINs (Figure 14). Thermal damage was also evidenced by a blackened fabric surface and distortion of the net structure (Figures 9 and 10). Thus, thermal damage will not always be associated with hole formation depending on the exposure time and proximity of the net to the heat source. Both PET and PE are thermoplastic polymers with different melting points: PET ca. 260°C and PE ca. 120–135°C depending on grade [34]. The different thermal properties of the two materials may explain differences in the proportion of LLIN defects attributable to thermal damage (Figures 13 and 14). The burning behaviour of textile materials is influenced by factors such as the nature of the ignition source, time of contact, the fabric orientation and point of ignition (e.g. at the edge or face of the fabric, or top or bottom), the ambient temperature and relative humidity, the air velocity and fabric structure [35].

The nature and extent of structural damage in a LLIN, whether or not associated with the formation of holes will depend upon the agencies of wear that it is exposed to during use, which is in turn likely to reflect local environmental conditions and modes of use. In some localities, rodent infestation is believed to be a source of hole formation in LLINs [22], and there may be others that have not yet been identified. It is reasonable to assume that the degree of damage accumulated by LLINs will depend heavily on how they are treated and used by their owners. This implies that social, behavioural and possibly economic factors may be important. The LLINs in the present study were obtained from more than one village but nevertheless, the same basic damage mechanisms were consistently observed. The present study was a preliminary evaluation of a small sample of LLINs (n = 100) from one region of Ghana, and therefore the experimental findings cannot be generalised.

A large scale, comprehensive study of the nature and specific causes of structural defects in LLINs retrieved from the field in different locations is required if a detailed understanding of the full range of factors that lead to hole formation is to be developed. This detailed understanding is an essential pre-requisite to enable improved longer-lasting nets to be designed and developed in the future.

To minimize hole formation, the resistance to initial yarn breakage, successive breakages and unravelling of yarns in LLIN’s has to be increased. Filament breakage can quickly lead to defects large enough to undermine the physical barrier that is provided by the fabric to the passage of mosquitoes. In practice, the extent to which breakage, tearing and unravelling can be resisted will be influenced by factors such as polymer composition and filament tensile properties, yarn construction, knitting pattern (which relates to the geometrical arrangement and intermeshing of the constituent yarns) and surface coatings. To minimize hole formation as a result of thermal damage LLINs should not be used in close proximity to candles or naked flames or high temperature heat sources of any kind.

## Conclusions

In this preliminary study involving a small sample of used PET and PE LLINs (n = 100) obtained from Ghana, holes were initiated by various forms of mechanical and thermal damage. In terms of mechanical damage, breakage of filaments by blunt transverse pressure (ductile fracture) as well as by sharp transverse pressure (cutting) was observed. Polymer melting due to high temperature exposure was found to be an additional but less frequent mechanism of fabric distortion and hole formation. The physical interaction between LLINs and external solid objects commonly leads to snag damage that produces distortions in the fabric structure and/or protrusion of looped filaments on the fabric surface. Although these snags result from localised mechanical stress, no strong correlation could be found between snag frequency and hole formation suggesting that pre-formed snags may not always lead to holes by propagation. However, snags are indicative of mechanical damage in LLINs and if during use a rough or sharp object catches the LLIN and the applied forces are such that multiple yarns are broken, a hole will result. A strong correlation between the bursting strength and the number of holes in used LLINs could not be confirmed, supporting the suggestion that bursting strength may not be a robust indicator of the durability of LLINs under field conditions if used as a sole parameter.
